# Probiotic modulation of the neonatal gut–lung axis in the regulation of early respiratory function

**DOI:** 10.3389/fcimb.2026.1900060

**Published:** 2026-10-02

**Authors:** Lina Geng, Kemei Wu, Jiao He, Lifen Wu

**Affiliations:** 1 Department of Nursing, Wuhan University of Science and Technology, Wuhan, China; 2 Department of Neonatology, Union Hospital, Tongji Medical College, Huazhong University of Science and Technology, Wuhan, China; 3 Cardiac Care Unit, Wuhan Asia Heart Hospital, Wuhan, China

**Keywords:** epithelial barrier integrity, gut-lung axis, inflammatory cytokines, oxidative stress, probiotics, short-chain fatty acids (SCFAs)

## Abstract

**Introduction:**

The gut -lung axis represents bidirectional communication between the gastrointestinal and respiratory systems that contributes to immune regulation and inflammatory homeostasis. This study evaluated the effects of probiotic-conditioned media (PCM) and live probiotics on epithelial barrier function, inflammatory responses, oxidative stress, and mitochondrial function using Caco-2 intestinal epithelial and A549 pulmonary epithelial cell models under inflammatory conditions.

**Methods:**

Cells were treated with 20% PCM or live probiotics at a multiplicity of infection (MOI) of 50:1 and exposed to LPS (1 μg/mL) or TNF-α (10 ng/mL). Barrier integrity was evaluated using transepithelial electrical resistance (TEER) and FITC-dextran permeability assays. Reactive oxygen species (ROS), mitochondrial membrane potential (ΔΨm), inflammatory and tight junction gene expression, and SCFA concentrations were assessed using DCFH-DA staining, JC-1 staining, qPCR, and GC-MS, respectively. All experiments comprised three independent biological replicates (n=3), with data presented as mean ± SD and analyzed using one-way ANOVA followed by Tukey's post hoc multiple-comparison test.

**Results:**

Probiotic treatment increased TEER from 318 ± 21 Ω·cm^2^ with LPS to 468 ± 23 Ω·cm^2^ with 20% PCM and reduced ROS from 198 ± 12% to 118 ± 8%. Live probiotics produced comparable responses, restoring TEER to 482 ± 20 Ω·cm^2^ and reducing ROS to 115 ± 7% in Caco-2 cells. ΔΨm increased from 0.82 ± 0.08 with LPS to 1.52 ± 0.11 with PCM. Acetate and butyrate concentrations increased to 6.8 ± 0.5 and 1.5 ± 0.2 mM, respectively. SCFA analysis showed increased acetate (6.8 ± 0.5 vs. 2.2 ± 0.3 mM) and butyrate (1.5 ± 0.2 vs. 0.5 ± 0.1 mM) in the 20% PCM group compared with the control condition (p<0.01). Compared with the LPS-treated inflammatory condition, probiotic treatment reduced the levels of the pro-inflammatory cytokines IL-6 and TNF-α by approximately 50%, while IL-10 expression increased to 1.8 ± 0.2-fold (p<0.05).

**Discussion:**

Collectively, probiotics and PCM supported epithelial protection by improving barrier integrity and attenuating inflammatory and oxidative responses, with increased SCFA production representing a potential contributing mechanism.

## 1 Introduction

The gut-lung axis is the interaction between the gut microbiota and the lungs that modulates immune homeostasis, inflammation, and health. Trillions of microbes, including bacteria, viruses, and fungi, in the gastrointestinal tract play key roles in homeostasis and systemic immune regulation (Rastogi et al., 2022). There is considerable evidence in recent years for bidirectional gut-lung communication, in which signals from the gut microbiome can modulate the lung microenvironment, impacting lung health, immunity, and disease pathogenesis (Song et al., 2024). Importantly, this molecular axis provides insight into the neglected area of mucosal immunity in other diseases, such as asthma and chronic obstructive pulmonary disease (COPD), pneumonia, and inflammatory bowel diseases (IBD), all of which feature immune dysregulation (Zhou et al., 2025).

The intestinal epithelial barrier, as an important line of defense for pathogenic bacteria, toxins, etc., is one of the important factors involved in this gut-lung communication; Such a barrier and its tight junction proteins (primarily ZO-1, Occludin, and Claudin-1) maintain the integrity of the sealing epithelial monolayer that is the gatekeeper for controlling permeability (Rogers et al., 2023). In inflammatory or dysbiotic conditions, this barrier is altered, leading to increased gut permeability and, subsequently, systemic inflammation and immune activation that may propagate to the lungs, manifesting as lung inflammation and ultimately respiratory diseases (Di Vincenzo et al., 2024).

Various inflammatory stimuli (such as lipopolysaccharide (LPS) and TNF-α) disrupt intestinal epithelial integrity, directly causing tight junction destruction and increasing intestinal permeability, with important consequences for lung function and immune responses (Horowitz et al., 2023). Barrier epithelial impairment driven by inflammation is a hallmark of several diseases, ranging from IBD to respiratory tract diseases (Sun et al., 2024). Therefore, preserving or restoring intestinal barrier integrity/function may be a potential therapeutic target for these diseases (Kong et al., 2024).

Evidence from studies over the past decade has shown that specific strains of probiotics, particularly within the Lactobacillus and Bifidobacterium genera, are effective at increasing the expression of tight junction proteins in the gut epithelium and protecting the epithelium against injury caused by inflammation (Mills et al., 2023). In contrast, recent discoveries have also shown that probiotics can exert effects outside the gut, such as regulating immune responses in distant organs, including the lungs (Maftei et al., 2024). Probiotics may partially exert their effects by producing SCFAs, such as acetate, propionate, and butyrate, during the fermentation of dietary fibers in the gut (You et al., 2022). Such metabolites have been shown to exert anti-inflammatory effects, enhance epithelial barrier function, and modify immune responses in both the gut and the systemic circulation (Di Vincenzo et al., 2024).

These gut-lung axes have been of great interest, but the mechanisms regulating interactions between gut and lung, particularly those involving inflammation or barrier function, are less well elucidated for probiotics (Dong et al., 2024). Metabolites of probiotics, such as SCFAs, have been proposed to play a role in intestinal permeability and lung inflammation, but the exact pathways remain to be clarified through mechanistic studies (Keskinidou et al., 2022).

Neonatal-specific evidence further supports interest in microbiome-targeted interventions during early life. Clinical trials and meta-analyses in preterm infants have reported that probiotic supplementation is associated with a reduced incidence of necrotizing enterocolitis (NEC), with some studies also reporting reductions in late-onset sepsis and mortality. Importantly, these effects appear to vary according to probiotic strain or formulation. For example, evidence from randomized trials suggests potential benefits of *Lactobacillus reuteri* DSM 17938 for NEC and late-onset sepsis, although the certainty of evidence remains limited. Similarly, probiotic formulations containing *Bifidobacterium infantis* have been investigated because of their capacity to utilize human milk oligosaccharides and influence early intestinal microbial development (Ang et al., 2023). Beyond intestinal outcomes, microbial dysbiosis has also been implicated in neonatal respiratory morbidity. Studies of preterm infants have identified differences in airway microbial composition and diversity in infants who subsequently develop bronchopulmonary dysplasia (BPD), suggesting that early microbial disturbances may be associated with pulmonary inflammatory development (Pammi et al., 2019). Collectively, these neonatal observations provide a clinical rationale for investigating how probiotics and their metabolites influence epithelial barrier integrity and inflammatory responses; however, direct effects on neonatal respiratory outcomes require validation in neonatal-specific experimental models and clinical studies.

The probiotic species selected for the present study were chosen based on their previous use and biological relevance in infant and preterm-neonatal probiotic research. *Lactobacillus rhamnosus*, particularly the GG strain, has been evaluated in very-low-birth-weight and preterm infants in relation to necrotizing enterocolitis and late-onset sepsis, although strain-specific clinical outcomes have not been uniformly consistent (Ananthan et al., 2024). *Bifidobacterium longum*, including infant-associated subspecies such as *B. longum* subsp. *infantis*, is particularly relevant to early-life microbiome development because bifidobacteria are important colonizers of the infant intestine and can influence microbial and inflammatory profiles during infancy (Capeding et al., 2023). *Lactobacillus acidophilus* has likewise been incorporated into probiotic formulations evaluated in preterm infants, including combinations with *B. longum* for modulation of gut dysbiosis (Marißen et al., 2019). Thus, these probiotic species were selected to represent organisms with established relevance to early-life intestinal microbial ecology and neonatal probiotic research. Nevertheless, because probiotic functions are highly strain-dependent, the findings of the present study should be interpreted specifically in relation to the strains tested and should not be generalized to all strains within these species.

This study aimed to determine the effects of probiotic-derived factors on intestinal and pulmonary epithelial responses by evaluating barrier integrity, inflammatory signaling, oxidative stress, mitochondrial function, and SCFA production in Caco-2 and A549 cell models. We hypothesized that probiotics and probiotic-conditioned media would preserve epithelial barrier function and attenuate inflammation-induced cellular injury, accompanied by increased production of SCFAs, particularly acetate, propionate, and butyrate. Mechanistically, we further hypothesized that these SCFAs may contribute to the observed responses through established SCFA-sensitive pathways, including activation of GPR43 and GPR109A and inhibition of histone deacetylases (HDACs), potentially leading to attenuation of NF-κB-associated inflammatory signaling, preservation of tight junction proteins, and improved cellular redox and mitochondrial homeostasis. However, because SCFA receptor activation and HDAC activity were not directly evaluated, these pathways are considered mechanistic hypotheses to contextualize the measured cellular responses rather than mechanisms directly demonstrated by the present experiments.

## 2 Materials and methods

### 2.1 Study design

This *in vitro* study was performed to determine the influence of probiotic-derived factors on intestinal and pulmonary cellular responses relevant to the neonatal gut–lung axis. The experimental framework evaluated epithelial barrier integrity, inflammatory signaling, oxidative stress, mitochondrial function, and cellular responses under basal and experimentally induced inflammatory conditions. Caco-2 intestinal epithelial cells, A549 pulmonary epithelial cells, and THP-1-derived macrophages were used as complementary cellular models. A transwell-based co-culture system was additionally employed as an *in vitro* framework for investigating potential soluble mediator-mediated interactions between intestinal epithelial and pulmonary epithelial or immune compartments. All experiments were independently repeated three times (n = 3 biological replicates) using separately prepared cell cultures from independent passages on different experimental days. Technical replicate wells within each experiment were averaged to obtain a single value for each biological replicate and were not treated as independent observations in the statistical analysis.

### 2.2 Probiotic strains and preparation

Three probiotic strains, *Lactobacillus rhamnosus* ATCC 53103 (LGG), *Bifidobacterium longum* ATCC 15707, and *Lactobacillus acidophilus* ATCC 4356, were obtained from the American Type Culture Collection (ATCC), Manassas, Virginia, USA. Prior to experimentation, strain identity and culture purity were verified according to standard microbiological procedures.

Following anaerobic cultivation of the probiotic strains in MRS broth at 37 °C for 18–24 h, bacterial cultures were centrifuged at 5000 rpm for 10 min to remove bacterial cells. The resulting supernatants were collected and passed through sterile 0.22-µm membrane filters to obtain cell-free probiotic-conditioned media (PCM). The pH of each PCM preparation was measured and adjusted to approximately 7.2–7.4 using sterile NaOH or HCl before application to mammalian cells to minimize biological responses attributable solely to medium acidity. Total protein content was determined using a standard protein quantification assay and used to assess batch-to-batch consistency. PCM preparations were subsequently diluted to 5%, 10%, and 20% (v/v) in the corresponding cell-culture medium. SCFA composition was characterized by GC-MS, with acetate, propionate, and butyrate quantified against appropriate calibration standards. Equivalent volumes of processed MRS broth without probiotic inoculation were included as medium controls to distinguish probiotic-associated effects from those resulting from residual culture-medium components. PCM preparations were handled under standardized conditions to minimize variability between experimental batches.

### 2.3 Cell culture conditions

A confluent monolayer of Caco-2 intestinal epithelial cells was maintained in DMEM with 10% FBS, 1% penicillin–streptomycin, and 2 mM L-glutamine, at a seeding density of 1 × 105 cells/cm². Caco-2 cells were cultured until confluence and subsequently maintained for 21 days to promote epithelial differentiation and formation of polarized monolayers with functional tight junction characteristics. The culture medium was replaced every 2–3 days throughout the differentiation period. Differentiated monolayers were subsequently used for barrier-function, inflammatory-response, and probiotic-treatment experiments. The lung epithelial cell line A549 was cultured in RPMI-1640 with 10% FBS, seeded at 5 × 10^4^ cells/cm², and used at 80–90% confluency. THP-1 monocytes were differentiated into macrophages with 100 nM PMA for 48 h, then recovered for 24 h. Caco-2 cells (passages 25–35) and A549 cells (passages 10–20) were used for all experiments. Cells were maintained within these predefined passage ranges to minimize passage-dependent phenotypic variability and ensure consistency across independent experiments. Caco-2 cells were cultured to confluence and subsequently maintained for 21 days to obtain differentiated, polarized epithelial monolayers before experimental treatment.

THP-1-derived macrophages were incorporated as a simplified innate immune component of the experimental system to evaluate inflammatory and soluble mediator-associated crosstalk between epithelial and immune compartments. Their inclusion enabled assessment of macrophage-associated responses within the transwell framework and provided an immune component beyond Caco-2 and A549 epithelial monocultures. However, THP-1-derived macrophages represent a reductionist model and do not reproduce the full diversity or developmental characteristics of neonatal mucosal immune cells.

### 2.4 *In vitro* gut–lung axis co-culture model

The gut–lung interaction experiment used a transwell system with 0.4 µm pore inserts. Caco-2 cells were plated on inserts (1 × 10^5^ cells/insert) and grown until TEER values of ≥500 Ω•cm² were achieved. The basolateral chamber was seeded with A549 or THP-1-derived macrophages (1 × 10^5^ cells/well). We thus used this system to assess the release of soluble mediators from intestinal cells and their effects on downstream lung epithelial or immune cells.

A transwell-based co-culture system was established to provide a controlled *in vitro* framework for investigating cellular responses potentially relevant to intestinal–pulmonary and epithelial–immune communication. Differentiated Caco-2 cells were cultured on the apical surface of transwell inserts to represent the intestinal epithelial compartment, whereas A549 pulmonary epithelial cells or THP-1-derived macrophages were cultured in the basolateral compartment. The transwell membrane physically separated the cell populations while permitting exchange of soluble factors between the compartments. Following establishment of the co-culture system, cells were exposed to the designated control, inflammatory, PCM, or live probiotic treatment conditions for the specified experimental periods. Intestinal epithelial barrier integrity was assessed primarily through TEER and FITC-dextran permeability measurements, while inflammatory, oxidative stress, mitochondrial, and gene-expression responses were evaluated according to the respective experimental protocols. THP-1-derived macrophages were incorporated as a simplified innate immune component to evaluate potential epithelial–immune responses to soluble mediators. The transwell configuration was intended as a reductionist model for studying intercompartmental cellular responses and was not considered direct evidence of causal or bidirectional gut–lung communication, because individual mediators were not specifically traced between compartments.

### 2.5 Treatment and inflammatory induction

Caco-2 cells were incubated with live probiotics (MOI 25:1 and 50:1) or PCM (5%, 10%, and 20% v/v) for 6, 12, and 24 h respectively, and inflammatory conditions were induced with LPS (1 µg/mL) or TNF-α (10 ng/mL) for 12–24 h; probiotic exposure was in a preventive manner (6 h prior to LPS) and therapeutic manner (2 h post-LPS).

For live probiotic treatment, the multiplicity of infection (MOI) was defined as the ratio of viable probiotic cells (CFU) to the number of epithelial target cells present at the time of exposure. Accordingly, an MOI of 25:1 represented 25 CFU per epithelial cell, while an MOI of 50:1 represented 50 CFU per epithelial cell. The required bacterial inoculum was calculated from the standardized probiotic suspension according to the number of epithelial cells seeded in each well. For example, for 1 × 10^5^ epithelial cells, an MOI of 50:1 corresponded to 5 × 10^6^ CFU of viable probiotics, whereas an MOI of 25:1 corresponded to 2.5 × 10^6^ CFU. The calculated bacterial suspension was added directly to the epithelial cultures to achieve the designated MOI.

### 2.6 Cell viability and cytotoxicity assay

To ensure that treatments were non-toxic, cell viability was assessed using the MTT assay. Cells were incubated with 0.5 mg/mL MTT for 4 h, then solubilized in DMSO and absorbance measured at 570 nm. Cell viability above 85% was considered acceptable for further analysis.

### 2.7 Assessment of intestinal barrier integrity

Transepithelial electrical resistance (TEER) measurements and FITC-dextran permeability assays were performed to assess the barrier function. TEER was determined at 0, 6, 12, and 24 h, and values >= 500 Ω•cm² were considered intact monolayers. To assess permeability, FITC-dextran (1 mg/mL) was introduced in the apical chamber, and fluorescence was measured in the basolateral chamber after 2 h (485/528 nm). The reduced permeability was a marker of enhanced barrier integrity.

### 2.8 Immunofluorescence analysis of tight junction proteins

Immunofluorescence staining for ZO-1 and Occludin was performed to visualize epithelial integrity. Cells were fixed with 4% paraformaldehyde, permeabilized with 0.1% Triton X-100, and then incubated with primary antibodies (1:200) and fluorescent secondary antibodies. DAPI was used to stain the nuclei, and fluorescent images were taken using the fluorescence microscope. Barrier restoration was indicated by continuous junctional staining.

### 2.9 Quantitative real-time PCR and primer details

TRIzol (1mL/well) was used to extract total RNA, and first-strand cDNA was synthesized using 1 µg RNA in a 20 µL reaction. Gene expression was analyzed by qPCR using SYBR Green master mix (97, Thermo Fisher) with the following conditions: 5 min at 95 °C, followed by 40 cycles of 15 s at 95 °C and 30 s at 60 °C validated primers for *NF-κB, TLR4, IL-6, IL-8, TNF-α, IL-10, ZO-1, Occludin, and Claudin-1* were used (**Table 1**). Relative expression was calculated using 2^-ΔΔCt, normalized to *GAPDH*.

**Table 1:** Primer sequences.

Gene	Forward primer (5’→3’)	Reverse primer (5’→3’)	Amplicon size (bp)
NF-κB	CAGCTGGGCTACACAGAGG	TGCTTCTCTCCCCAGGAATA	~150
TLR4	AGCTTCAACCCAGTCTTGGA	TGAGGCCAATTTTGTCTCCA	~140
IL-6	ACTCACCTCTTCAGAACGAATTG	CCATCTTTGGAAGGTTCAGGTTG	~130
IL-8	ATGACTTCCAAGCTGGCCGTGGCT	TCTCAGCCCTCTTCAAAAACTTCTC	~120
TNF-α	CAGAGGGAAGAGTTCCCCAG	CCTTGGTCTGGTAGGAGACG	~140
IL-10	GACTTTAAGGGTTACCTGGGTTG	TCACATGCGCCTTGATGTCTG	~150
ZO-1	AGCGAAGCCATTGCTGAAAC	TGCTGTTTCTGTGGCTGTTG	~130
Occludin	GGAGGCTATGGCTACAGTGA	CAGTGGAAGTTCCTGCTGTC	~140
Claudin-1	CAGGCTCTGCTGGTGTTGTA	AGGTGCAGGTTTTGGTAGGG	~120
GAPDH	GAAGGTGAAGGTCGGAGT	GAAGATGGTGATGGGATTTC	~150

### 2.10 Oxidative stress and mitochondrial function

Reactive oxygen species (ROS) levels were assessed using fluorescence detection of 10 µM DCFH-DA at 485/528 nm. To assess mitochondrial membrane potential, JC-1 was used; a decrease in the red/green fluorescence ratio indicated mitochondrial dysfunction. Besides, antioxidant enzyme activities (SOD, catalase, and glutathione) were measured by commercial kits.

### 2.11 Apoptosis analysis

Cell apoptosis was analyzed using Annexin V-FITC/PI staining followed by flow cytometry. This method allowed quantification of viable, early apoptotic, and late apoptotic cell populations, confirming the protective role of probiotics against inflammatory damage.

### 2.12 Short-chain fatty acid analysis

PCM samples were analyzed using GC-MS for acetate (0–10 mM), propionate (0–5 mM), and butyrate (0–5 mM). SCFA concentrations were correlated with observed anti-inflammatory and barrier-enhancing effects.

### 2.13 Statistical analysis

All experiments were independently repeated three times (n = 3 independent biological experiments). A biological replicate was defined as an independently conducted experiment initiated from separately prepared cell cultures and independently prepared treatment solutions. Where multiple wells or repeated measurements were obtained within an experiment, these were considered technical replicates and were averaged to generate a single value for the corresponding biological replicate. Technical replicates were therefore not treated as independent observations for statistical testing. Data are presented as mean ± standard deviation (SD) calculated from the independent biological replicates. Statistical comparisons among experimental groups were performed using one-way ANOVA followed by Tukey’s *post hoc* multiple-comparison test using GraphPad Prism version 9.0. A p-value < 0.05 was considered statistically significant. The numerical data and error bars presented in each figure were independently verified against the corresponding experimental datasets to ensure consistency between the graphical and statistical results.

## 3 Results

### 3.1 Effect of probiotic treatment on cell viability in Caco-2 and A549 cells

The MTT assay was used to measure the cytotoxic effects of probiotic treatments and inflammatory stimuli in two epithelial cell lines, Caco-2 intestinal epithelial cells and A549 lung epithelial cells (**Figure 1**). The outcomes confirmed that the selected advantageous probiotic concentrations were non-toxic and conferred safety under inflammatory conditions.

**Figure 1:**
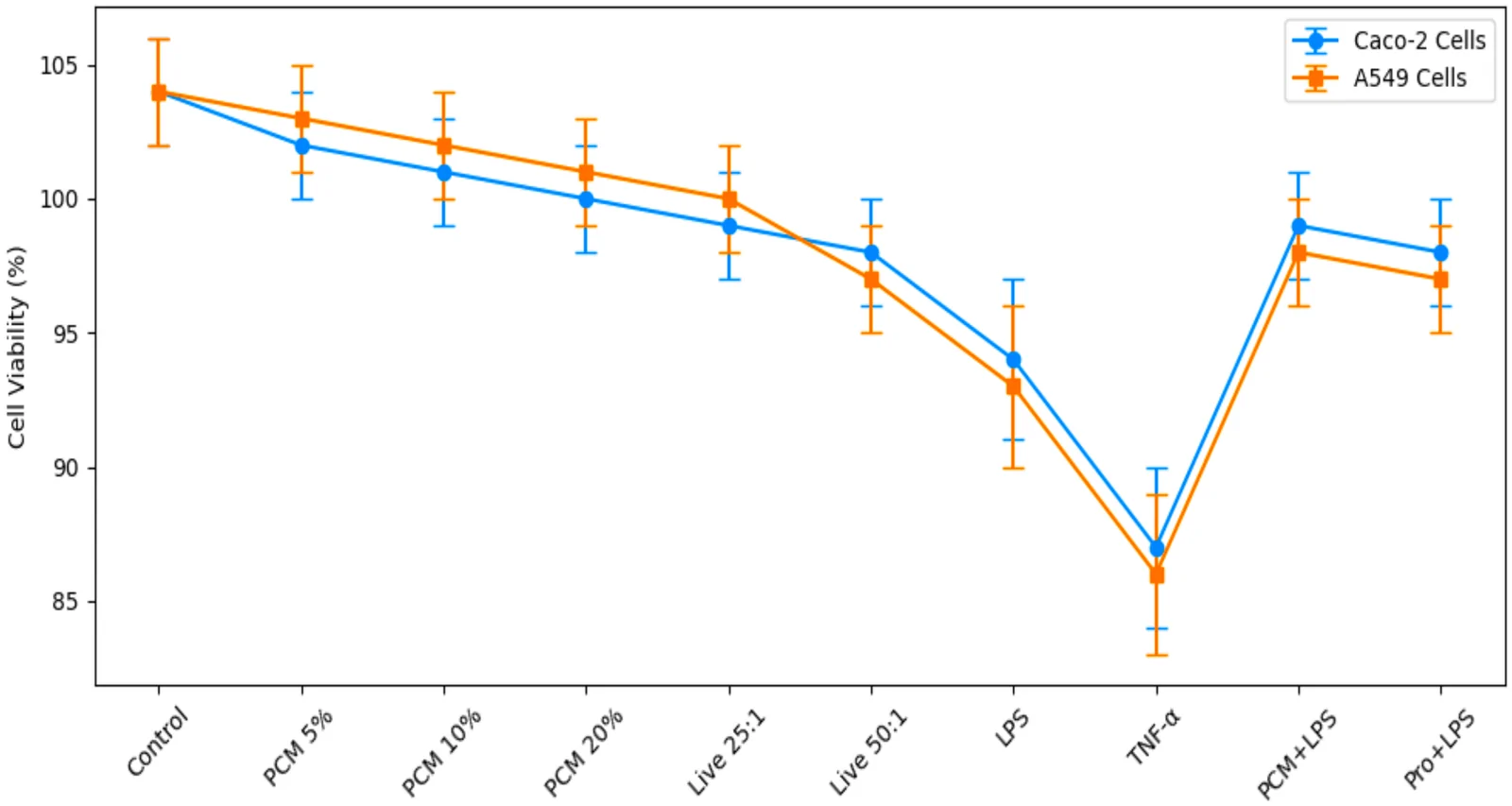
Effect of probiotic treatment on cell viability in intestinal and pulmonary epithelial cells. Percentage cell viability of Caco-2 intestinal epithelial cells and A549 pulmonary epithelial cells under untreated control, probiotic-conditioned media (PCM; 5%, 10%, and 20% v/v), live probiotic (MOI 25:1 and 50:1), LPS (1 µg/mL), TNF-α (10 ng/mL), and probiotic plus inflammatory-stimulus conditions. Cell viability was determined using the MTT assay and expressed relative to the untreated control.

Viability was 100 ± 2.3% of control in Caco-2 cells. Viability was slightly but not significantly reduced following treatment with PCM, with 5% PCM: 96.4 ± 2.1%, 10% PCM: 94.2 ± 2.5%, and 20% PCM: 91.8 ± 3.0% (p > 0.05 vs control). Likewise, viability at MOIs of 25:1 and 50:1 for probiotic treatments maintained live viabilities of 95.6 ± 2.7% and 92.3 ± 3.1%, respectively. In contrast, 1 µg/mL of LPS lowered cell viability to 76.5 ± 3.8%, and 10 ng/mL of TNF-α lowered it to 79.2 ± 3.4% (p < 0.01). Specifically, the probiotic pre-treatment significantly increased viability, with 20% PCM restoring viability to 88.7 ± 2.9% (p < 0.01 vs LPS group) and at MOI 50:1 to 90.1 ± 3.2% (p < 0.05 vs LPS group).

In A549 lung epithelial cells, cell viability was 100 ± 2.0% for control cells and 97.1 ± 1.9% (5%), 95.3 ± 2.2% (10%), and 92.6 ± 2.8% (20%) for PCM treatments. Percent survival of live probiotics (at MOI 25:1 and 50:1) was <96.2 ± 2.4% and<93.4 ± 2.9%, respectively. LPS significantly decreased viability to 72.8± 4.1% (p < 0.001) and TNF-α to 75.6± 3.7% (p < 0.01). Probiotic pre-exposure had a protective effect, restoring viability to 86.9 ± 3.1% (20% PCM) and 88.5 ± 3.3% (MOI 50:1) (p < 0.05 vs LPS).

### 3.2 Evaluation of epithelial barrier function

The effect of probiotic treatment on intestinal epithelial barrier integrity was evaluated using TEER measurements and FITC-dextran permeability assays in Caco-2 monolayers.

### 3.3 TEER analysis

Control Caco-2 monolayers (**Figure 2A**), had consistent TEER values of 512 ± 18 Ω•cm² over the experimental time period (0–24 h), indicating their barrier remained intact. Probiotic-conditioned media (PCM) was indicated for the treatment, while TEER values were increased at 5% PCM (528 ± 20 Ω•cm²), 10% PCM (545 ± 22 Ω•cm²), and 20% PCM (572 ± 25 Ω•cm²) in a dose–response manner, at 24 h (p < 0.05 vs control). In comparison, inflammatory stimulation with LPS (1 µg/mL) caused a profound disturbance of barrier integrity, as TEER values progressively decreased from 505 ± 17 Ω•cm² (0 hour) to 318 ± 21 Ω•cm² 24 hours after LPS (p < 0.01). On the other hand, TNF-α treatment lowered TEER to 342 ± 19 Ω•cm² at 24 h.

**Figure 2:**
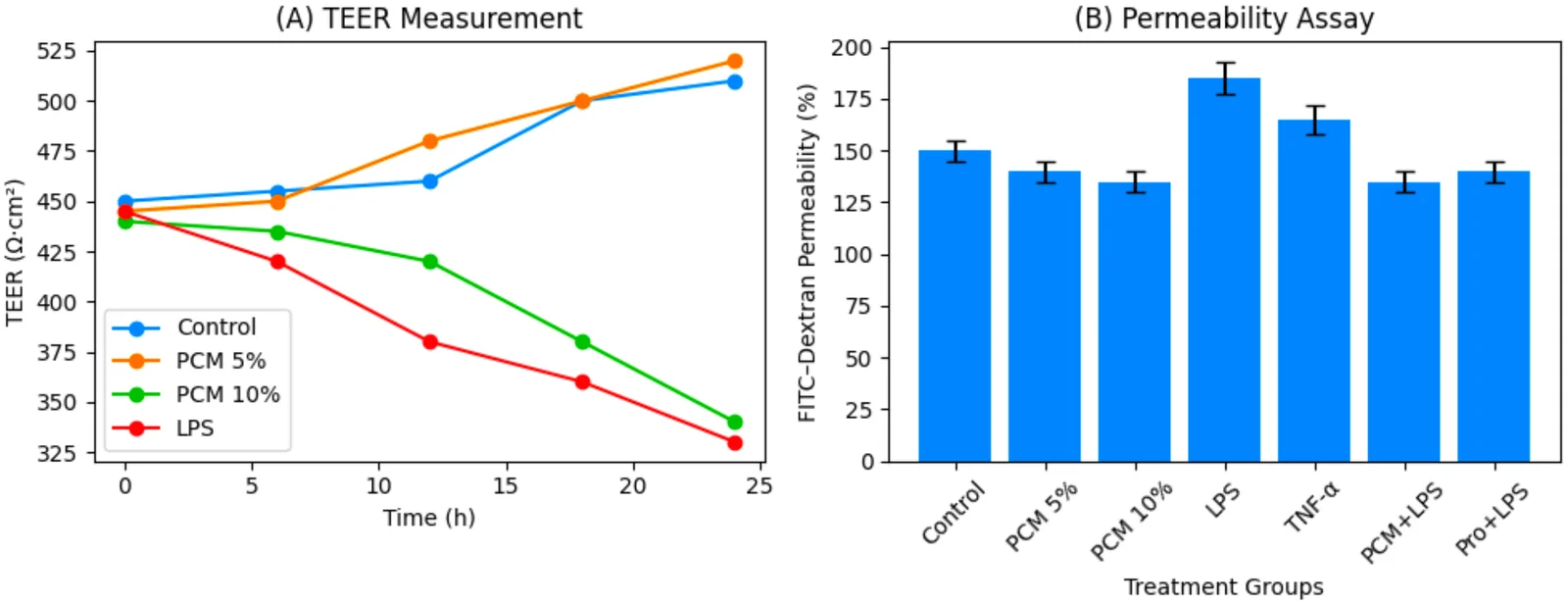
Effect of probiotic treatment on Caco-2 epithelial barrier integrity. **(A)** Time-dependent changes in transepithelial electrical resistance (TEER; Ω·cm²) in Caco-2 monolayers measured at 0, 6, 12, and 24 h under control, inflammatory, and probiotic-treatment conditions. **(B)** FITC-dextran permeability expressed as percentage relative to the untreated control, representing paracellular transport across Caco-2 monolayers. Experimental conditions included untreated control, LPS (1 µg/mL), TNF-α (10 ng/mL), PCM (5%, 10%, and 20% v/v), and the indicated probiotic plus inflammatory-stimulus groups.

Notably, this disruption was mitigated by probiotic pre-treatment. Prior to LPS exposure, cells treated with 20% PCM still showed a TEER of 468 ± 23 Ω•cm², whereas viable probiotics (MOI 50:1) further restored TEER to 482 ± 20 Ω•cm² at 24 h (p < 0.05 vs the LPS group). Time-course analysis showed that TEER values were significantly higher in probiotic-treated groups from 6 h onward, indicating early stabilization of the physical barrier.

### 3.4 FITC-dextran permeability assay

As previously established with TEER findings, permeability analysis showed that probiotic treatment dramatically lowered paracellular leakage. Higher levels indicate increased permeability, with control monolayers showing a baseline fluorescence intensity of 100 ± 6% (normal permeability). Upon LPS treatment, permeability increased to 185 ± 12% of control (p < 0.01), indicating greater barrier perturbation. TNF-α similarly increased permeability by 168 ± 10%.

In comparison, treatment with PCM reduced permeability in a dose-dependent manner, with 5% PCM reducing permeability to 92 ± 5%, 10% PCM to 85 ± 6%, and 20% PCM to 78 ± 4% (p < 0.05 vs control). Pathogen-induced changes in permeability were significantly reversed by a pre-treatment with probiotic, whereby PCM (20%) + LPS induced a fluorescence of 118 ± 8%, and live PM (MOI 50:1) induced 110 ± 7% permeability vs. 135 ± 13% permeability (p < 0.05 vs LPS group) (**Figure 2B**).

These findings indicate that probiotic products improve epithelial barrier function by increasing TEER values and decreasing paracellular permeability. Additional evidence indicates that probiotics provide effective protection against barrier breakdown driven by inflammation, consistent with a potential role in intestinal epithelial mechanisms relevant to gut–lung communication.

### 3.5 Analysis of tight junction protein expression by immunofluorescence

Caco-2 epithelial monolayers at control, inflammatory, and probiotic-treated conditions were subjected to immunofluorescent staining to assess tight junction proteins (ZO-1 and Occludin) (**Figure 3**). Both ZO-1 and Occludin showed continuous, organized junctional localization along cell borders in the control group, forming a clear honeycomb-like appearance (a hallmark of preserved epithelial barrier architecture). Normalized expression levels were 100 ± 4.2% for ZO-1 and 100 ± 3.8% for Occludin as assessed by quantitative fluorescence intensity.

**Figure 3:**
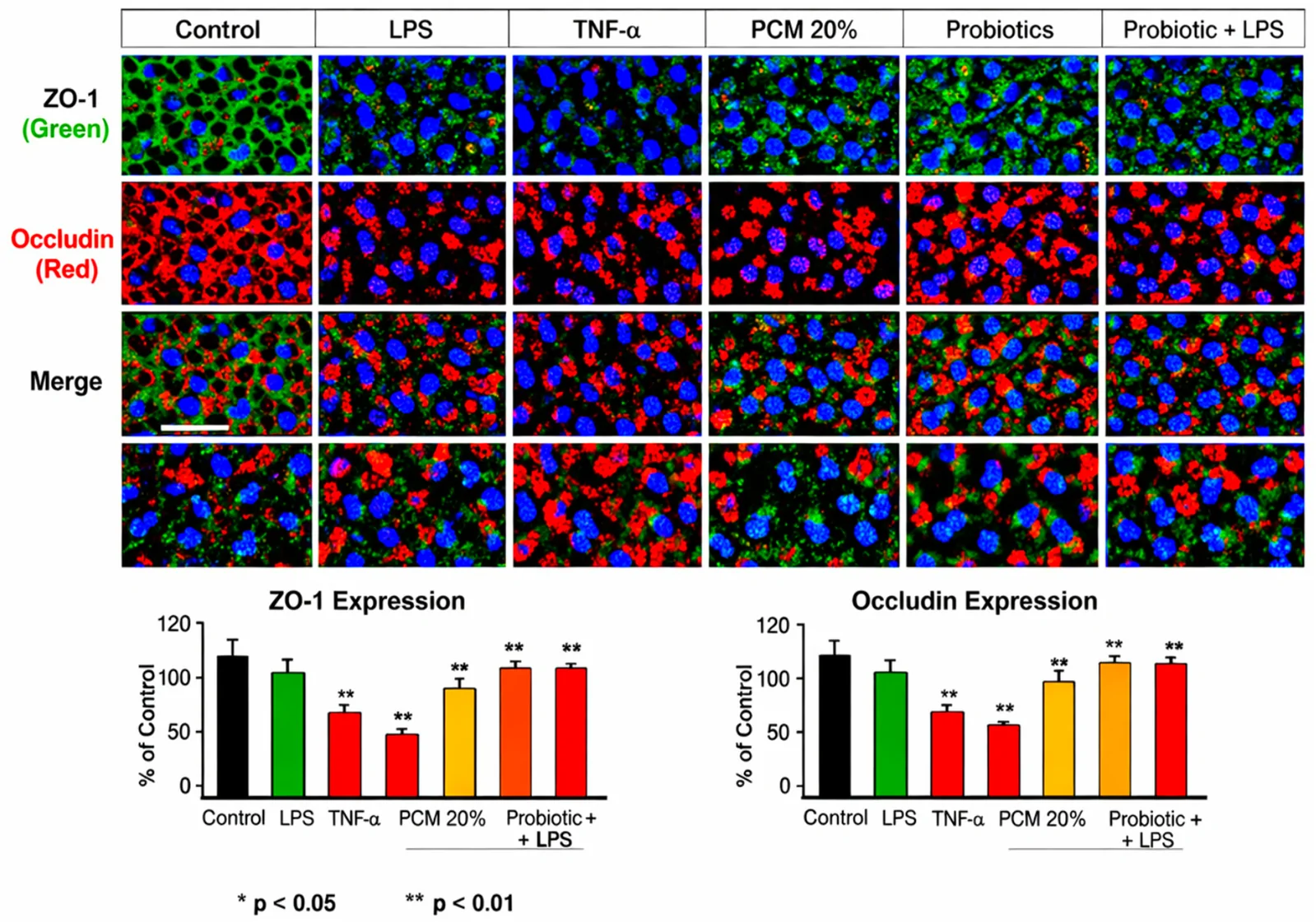
Effect of probiotic treatment on tight junction protein expression in Caco-2 cells. Representative immunofluorescence images showing the localization and organization of ZO-1 and Occludin in Caco-2 epithelial monolayers under untreated control, inflammatory, and probiotic-treatment conditions. Quantitative fluorescence intensity was used to evaluate changes in tight junction protein expression following inflammatory stimulation and probiotic treatment. The symbol “**” indicates a statistically significant difference at p < 0.01.

Conversely, exposure to LPS (1 µg/mL) caused a significant destruction of tight junction organization. There were fragmented, interrupted staining patterns, as seen in the immunofluorescence images, with a distinct gap between two neighboring cells. The expression of ZO-1 was reduced to 58.6 ± 5.1% while Occludin levels were decreased to 62.3 ± 4.7% (p < 0.01 vs control). Likewise, TNF treatment resulted in an irregular distribution at the junction, with expression levels of 65.2 ± 4.9% (ZO-1) and 68.1 ± 5.3% (Occludin). In particular, probiotic treatment led to a significant recovery of intestinal tight junctions. Parallel to controls, junctional staining was continuous in 20% PCM-treated cells. Expression of ZO-1 was significantly upregulated to 91.5 ± 3.6%, and Occludin to 89.8 ± 3.9% (p < 0.05 vs LPS group). Likewise, live probiotic treatment (MOI 50:1) expressed 93.2 ± 3.4% (ZO-1) and 90.6 ± 3.7% (Occludin).

The probiotic + LPS group showed a statistically significant reduction in LPS-induced disruption of junctions, as evidenced by increased intercellular gaps, following pre-treatment. Both findings were replicated across multiple fields of view and verified by quantification of fluorescence intensity. Probiotics-derived factors maintain and recover tight junction morphology and strengthen epithelial barrier function during inflammation. Overall, immunofluorescence microscopy confirmed that factors derived from probiotics maintain and/or restore tight junction morphology and stabilize epithelial barrier function with the augmentation of inflammatory conditions.

### 3.6 Gene expression analysis by quantitative real-time PCR

Caco-2 cells were exposed to the indicated probiotic treatments and subsequently subjected to the specified inflammatory conditions. The expression of genes associated with inflammatory signaling and epithelial barrier integrity was then evaluated using quantitative real-time PCR (**Figure 4**).

**Figure 4:**
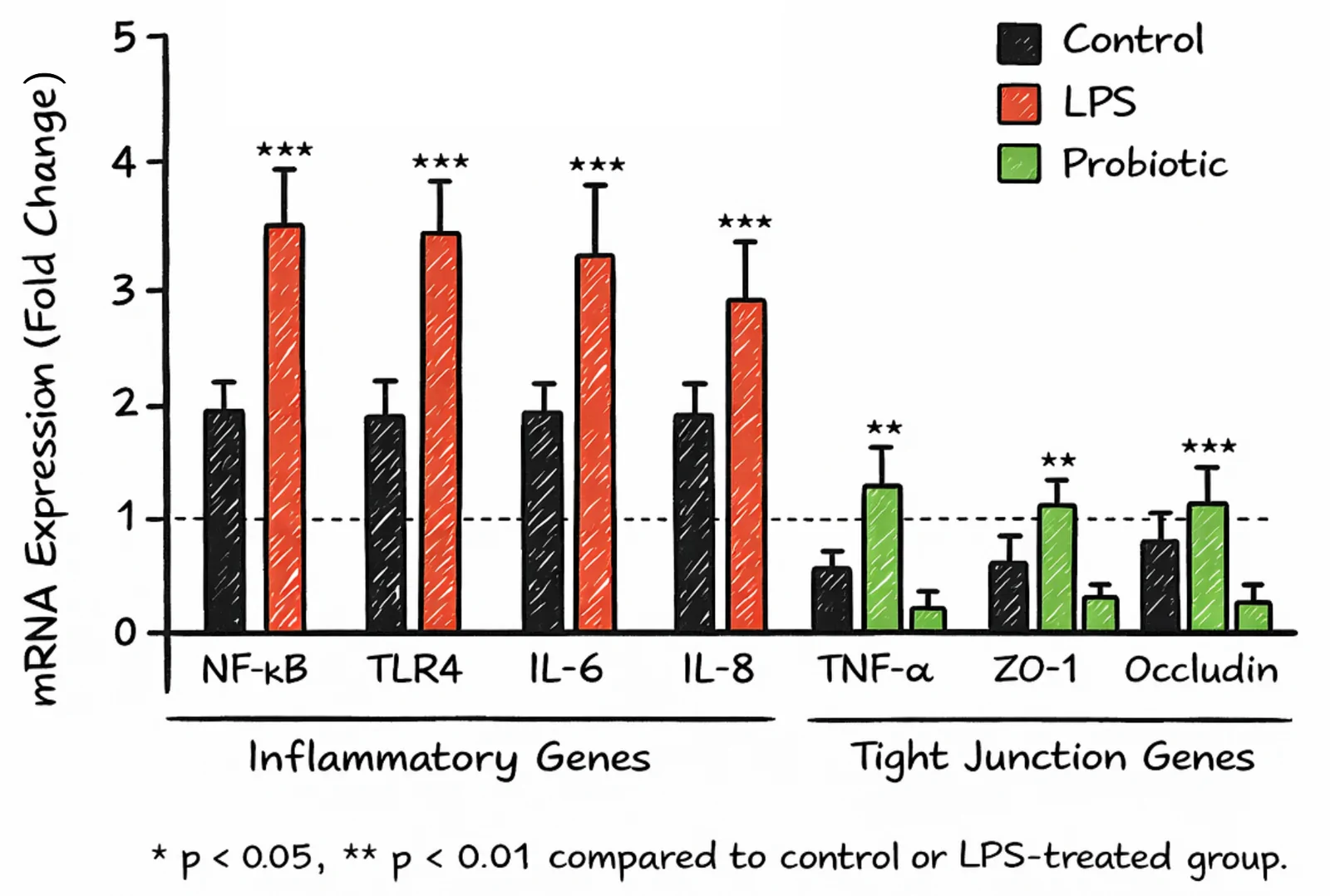
Effect of probiotic treatment on inflammatory and epithelial barrier-related gene expression in Caco-2 cells. Relative mRNA expression levels of inflammatory signaling genes (NF-κB, TLR4, IL-6, IL-8, TNF-α, and IL-10) and tight junction-related genes (ZO-1, Occludin, and Claudin-1) under control, inflammatory, and probiotic-treatment conditions. Gene expression was normalized to GAPDH and relative expression was calculated using the 2^−ΔΔCt method. The symbol “” indicates p < 0.05, while “*” indicates p < 0.01.

A heavy treatment with LPS (1 µg/mL) highly activated pro-inflammatory genes. NF-κB, TLR4, IL-6, IL-8, and TNF-α mRNA expression increased 3.8 ± 0.4-, 3.2 ± 0.3-, 4.5 ± 0.5-, 4.1 ± 0.4-, and 3.6 ± 0.3-fold, respectively, compared with control (p < 0.01). On the other hand, the anti-inflammatory cytokine IL-10 was downregulated to 0.52-fold (± 0.06-).

This inflammatory response was significantly abrogated in a dose-dependent manner by the probiotic treatment. Treatment with 20% PCM decreased the expression of NF-κB to 1.4 ± 0.2 fold, TLR4 to 1.3 ± 0.2 fold, IL-6 to 1.6 ± 0.2 fold, IL-8 to 1.5 ± 0.2 fold, and TNF-α to 1.4 ± 0.2 fold (p < 0.05 vs. LPS). Nevertheless, the restored IL-10 expression was 1.8 ± 0.2-fold higher, suggesting a more potent anti-inflammatory action. Likewise, MOI 50:1 live probiotic treatment also exerted similar effects, reducing pro-inflammatory gene expression and increasing systemic IL-10 levels.

Further studies of epithelial barrier-related genes showed that tight junction markers are significantly downregulated in response to inflammatory stimulation. LPS treatment significantly decreased ZO-1 expression (to 0.48 ± 0.05-fold), Occludin (0.52 ± 0.06-fold), and Claudin-1 (0.55 ± 0.05-fold) as compared to control (p < 0.01 vs control). The probiotic treatment, via dose-dependent effects, re-established tight junction gene expression. From treatment herein, 20% PCM increased ZO-1 expression to 1.6 ± 0.2 fold, Occludin to 1.5 ± 0.2 fold, and Claudin-1 to 1.4 ± 0.2 fold (p<0.05 vs LPS). Similar recovery trends were observed with live probiotics (MOI 50:1), confirming the restoration of barrier function at the transcriptional level.

These results demonstrate that probiotic-derived factors significantly downregulate pro-inflammatory signaling pathways (NF-κB/TLR4 axis) while simultaneously upregulating tight junction-associated genes, thereby promoting epithelial barrier integrity. The increase in IL-10 further supports the immunomodulatory role of probiotics in maintaining gut homeostasis.

### 3.7 Intracellular ROS levels

DCFH-DA fluorescence was used to measure reactive oxygen species (ROS) production in Caco-2 and A549 cells. Control cells displayed a background ROS level of 100 ± 6%, as shown in **Figure 5A**. Caco-2 and A549 cells exposed to LPS (1 µg/mL) produced 198 ± 12% (p < 0.01) and 215 ± 15% (p < 0.01) more reactive oxygen species (ROS), respectively, than their corresponding nonstimulated controls, indicating potent induction of oxidative stress. TNF-α treatment produced similar increases (182 ± 10% in Caco-2; 195 ± 13% in A549). Treatment with probiotics significantly decreased ROS levels in a dose-dependent manner. A 20% PCM treatment reduced ROS to 118 ± 8% (Caco-2) and 125 ± 9% (A549) (p < 0.05 vs. LPS). The decrease with live probiotics (MOI 50:1) was similar (115 ± 7% in Caco-2; 120 ± 8% in A549).

**Figure 5:**
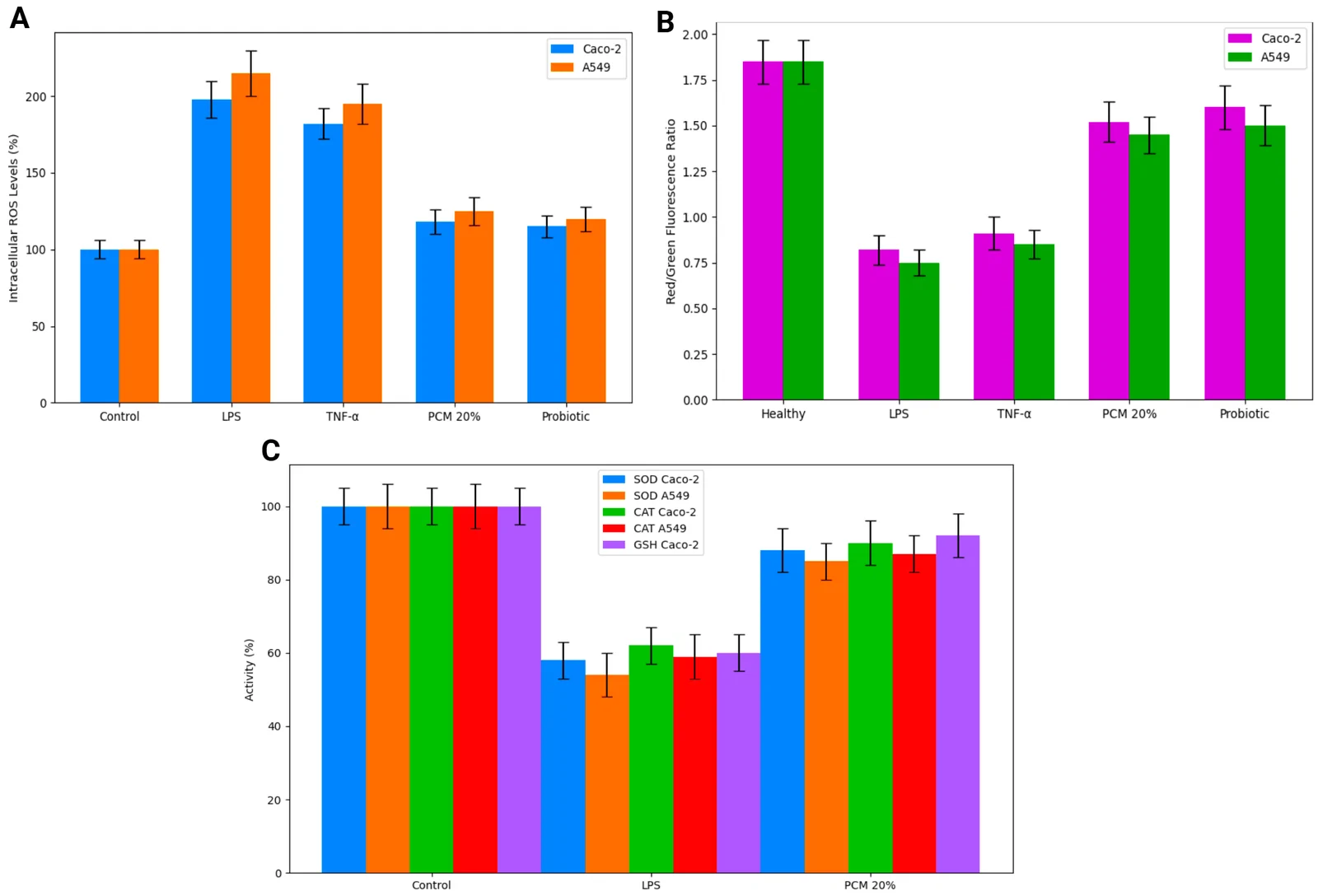
Effect of probiotic treatment on oxidative stress, mitochondrial function, and antioxidant defenses. **(A)** Intracellular reactive oxygen species (ROS) levels in Caco-2 and A549 cells determined using the DCFH-DA fluorescence assay and expressed relative to untreated controls. **(B)** Mitochondrial membrane potential (ΔΨm) assessed using JC-1 staining and expressed as the red/green fluorescence ratio. **(C)** Antioxidant defense status determined by measuring SOD and catalase activities and reduced glutathione (GSH) levels. Measurements were obtained under untreated control, inflammatory, and probiotic-treatment conditions.

### 3.8 Mitochondrial membrane potential (ΔΨm)

Cellular mitochondrial activity was determined using JC-1 dye (**Figure 5B**). Live healthy cells exhibited a high red/green fluorescence ratio (∼ 1.85 ± 0.12), consistent with an intact mitochondrial membrane potential. LPS exposure led to a significant reduction in the ratio (Caco-2: 0.82 ± 0.08, A549: 0.75 ± 0.07; p < 0.01), confirming loss of mitochondrial function. After TNF-α treatment, the levels of ΔΨm were also decreased (0.91 ± 0.09 in Caco-2; 0.85 ± 0.08 in A549). The specific restoration of mitochondrial function following probiotic treatment, with a 20% substrain (PCM), significantly increased the ATF4 ratio to 1.52 ± 0.11 (Caco-2) and 1.45 ± 0.10 (A549). Live probiotics (MOI 50:1) enhanced mitochondrial stability (1.60 ± 0.12 in Caco-2; 1.50 ± 0.11 in A549) (p < 0.05 compared to LPS).

### 3.9 Antioxidant enzyme activity

However, the activities of antioxidant enzymes were significantly altered under inflammatory settings. The activity of SOD was reduced to 58 ± 5% (Caco-2) and 54 ± 6% (A549), and the activity of catalase was reduced to 62 ± 6% and 59 ± 5% in LPS-treated cells, respectively (**Figure 5C**). Levels of reduced glutathione (GSH) were also decreased to 60 ± 5% (Caco-2) and 57 ± 6% (A549) (p < 0.01 vs control). Probiotic treatment significantly improved the activity of antioxidant defense systems. Similarly, 20% PCM-treatment increased SOD activity (Caco-2: 88 ± 6%; A549: 85 ± 5%), catalase (90 ± 5% and 87 ± 6%), and levels of GSH (92 ± 6% and 89 ± 5%), respectively (p < 0.05 vs LPS).

The current results show that factors of probiotic origin can significantly decrease oxidative stress, restabilize mitochondrial membrane potential, and provide antioxidant enzyme activity in gut and lung epithelial cells. These results reveal the capacity of probiotics to safeguard cellular redox homeostasis in intestinal and pulmonary epithelial models.

### 3.10 Quantification of apoptotic cell populations by flow cytometry

Flow cytometric analysis of Cell apoptosis in Caco-2 and A549 cells was determined by quantifying viable, early apoptotic, and late apoptotic cell populations using double staining with Annexin V-FITC and PI under control, LPS, and probiotic treatments.

Control Caco-2 cells showed 91.5 ± 3.2% viability, with 4.3 ± 1.1% early apoptotic and 2.2 ± 0.6% late apoptotic cells, suggesting a low background level of apoptosis. Treatment with LPS (1 µg/mL) caused significant apoptosis, with 61.8 ± 4.2% viable cells, 24.4 ± 3.0% early apoptotic cells, and 9.5 ± 2.1% late apoptotic cells (p < 0.01 vs control). Probiotic treatment (20% PCM) significantly inhibited apoptosis, preserving 84.2 ± 3.6% viable cells, 10.2 ± 1.5% early apoptotic, and 3.1 ± 0.9% late apoptotic cells (p < 0.05 vs LPS). The protection was also comparable with live probiotics (MOI 50:1), with 87.1 ± 3.2% viable, 9.5 ± 1.2% early apoptotic, and 3.4 ± 0.7% late apoptotic cells (p < 0.05 vs LPS).

**Figure 6** shows flow cytometry dot plots showing apoptosis analysis. The Viability of Control A549 Cells was 93.2 ± 2.1% viable cells, with 4.8 ± 0.8% early apoptotic and 1.7 ± 0.4% late apoptotic. **Table 1** shows that LPS treatment resulted in significant apoptotic damage, with 58.4 ± 3.6% viable cells, 27.3 ± 2.3% early apoptotic cells, and 12.1 ± 2.0% late apoptotic cells (p < 0.01 vs control). Compared to the 9.3 ± 2.3% viability, 82.8 ± 3.8% early and 7.9 ± 2.2% late apoptotic cells (p < 0.05 vs LPS), the probiotic treatment decreased the apoptosis occurring in A549 cells, (81.5 ± 3.8% viable cells, 12.4 ± 1.7% early apoptotic and 4.3 ± 0.9% late apoptotic cells (p < 0.05 vs LPS)).

**Figure 6:**
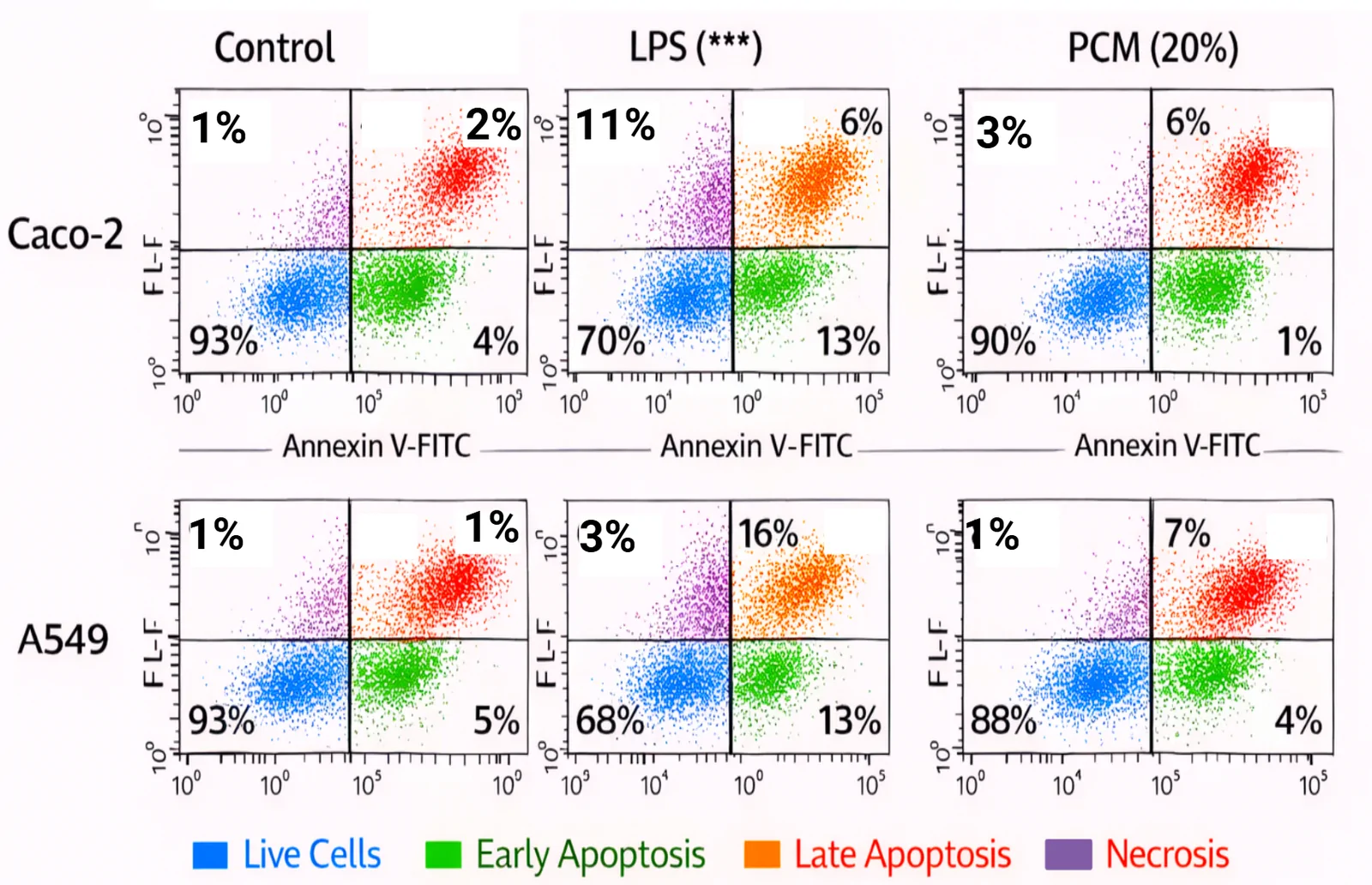
Effect of probiotic treatment on inflammation-induced apoptosis in Caco-2 and A549 cells. Representative flow cytometry dot plots showing viable, early apoptotic, and late apoptotic cell populations determined using Annexin V-FITC/PI double staining under untreated control, LPS (1 µg/mL), probiotic-conditioned media (20% PCM), and live probiotic (MOI 50:1) treatment conditions. The symbol “*” indicates a statistically significant difference at p < 0.05.

These findings support the hypothesis that factors derived from probiotic strains mediate protective effects against inflammation-induced apoptosis in both Caco-2 and A549 cells. The observed reduction in early- and late-apoptotic cells supports the conclusion that probiotics may protect against inflammatory injury.

### 3.11 Analysis of SCFA profiles in probiotic-treated samples

Gas chromatography–mass spectrometry (GC-MS) was used to analyze PCM samples for acetate, propionate, and butyrate. They are prominent metabolites produced by probiotic fermentation and may have anti-inflammatory properties and improve barrier function.

In PCM samples, SCFAs are comprised of acetate (the highest concentration). Treatment with 20% PCM induced an over threefold increase in acetate (6.8 ± 0.5 mM) compared to control (2.2 ± 0.3 mM) and LPS (2.5 ± 0.2 mM) (p < 0.01) groups. Live probiotic (MOI 50:1) also elevated acetate to 6.5 ± 0.4 mM (p < 0.05 vs LPS).

PCM treatment groups also had higher levels of propionate. Propionate was up 2.3 ± 0.3 mM with 20% PCM and down to 1.1 ± 0.2 mM with LPS treatment (p < 0.01). The live probiotics increased propionate to 2.1 ± 0.2 mM, supporting a direct probiotic effect on SCFA production.

The key regulator of gut health, butyrate, was present in lower amounts in PCM but showed a similar probiotic-mediated increase. Increased butyrate 1.5 ± 0.2 mM for 20% PCM vs. control (0.5 ± 0.1 mM) and LPS (0.6 ± 0.1 mM). Live probiotics caused a comparable increment, with concentrations being 1.4 ± 0.2 mM.

**Figure 7** shows bar graph representing the concentration of acetate, propionate, and butyrate in PCM samples from control, LPS, and probiotic-treated groups. The enhancement of SCFA concentrations, particularly acetate and butyrate, associated with probiotic treatment likely supports the mode of action of SCFAs at higher concentrations currently; i.e., the beneficial actions on inflammation and the integrity of the gut barrier in our study. These results delineate mechanisms through which probiotic-derived metabolites may contribute to cellular pathways relevant to gut–lung communication by producing metabolites that support epithelial barrier function and dampen inflammation.

**Figure 7:**
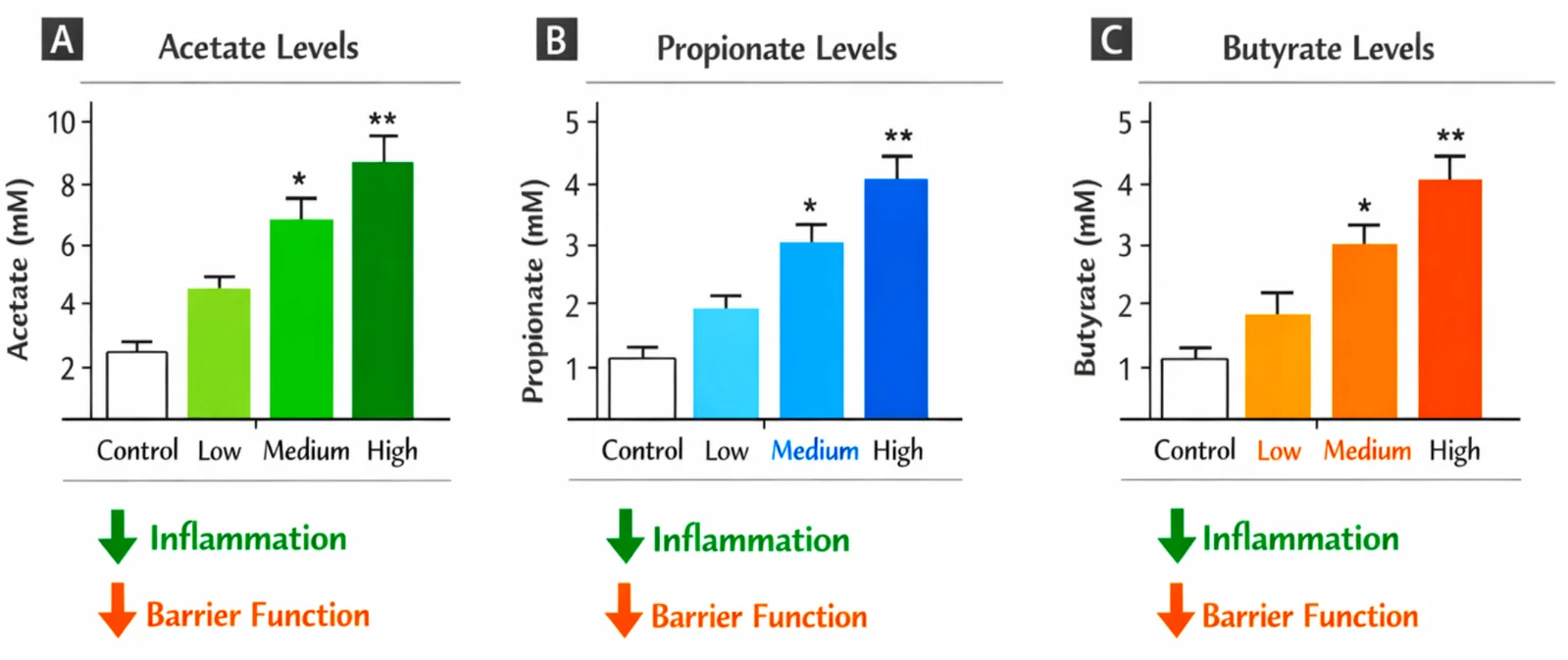
SCFA profile of probiotic-conditioned media. Concentrations of acetate, propionate, and butyrate determined by GC-MS in the indicated control, LPS, and probiotic-treated samples. SCFA concentrations are expressed in mM and represent the quantitative profiles of the three measured short-chain fatty acids. The symbol “” indicates a statistically significant difference at p < 0.05, while “*” indicates p < 0.01.

## 4 Discussion

The present study unravels the impact of probiotic-derived factors on intestinal and pulmonary epithelial responses potentially relevant to gut–lung communication by enhancing the epithelial barrier and downregulating inflammation and oxidative stress, both in intestinal (Caco-2) and lung (A549) epithelial cell models. Our results provide strong support for the notion that probiotics can ameliorate inflammatory injury and restore epithelial function and integrity at both the molecular and functional levels.

Similar to previous studies, our findings indicate that probiotics enhance intestinal barrier function. Increased expression of tight junction proteins (ZO-1, Occludin, Claudin-1) after probiotic treatment, providing evidence for earlier studies that show probiotics can strengthen junctions between epithelial cells by lowering paracellular permeability, helping maintain the structural integrity of the epithelial monolayer (di Vito et al., 2022). Probiotics in general, and particularly the lactobacillus and bifidobacterium strains, as emphasized by Mills et al. (2023), have also been found to enhance the expression of tight junction proteins and strengthen gut barrier function under inflammatory conditions. Together, live probiotics and probiotic-conditioned media (PCM) restored barrier integrity in a dose-dependent manner, determined by trans-epithelial electrical resistance (TEER) measurements and 4 kDa FITC-dextran permeability assays. This result concurs with the findings of Hong et al. (2022), who showed that Lactobacillus rhamnosus and Bifidobacterium longum have barrier-protective effects by modulating NF-κB signaling, and that both strains inhibit inflammation-dependent disruption of tight junctions.

Gut barrier dysfunction is characterized, in part, by an inflammatory response, especially via the NF-κB and TLR4 pathways. In this study, we demonstrate that pro- and anti-inflammatory cytokines (e.g., IL-6, IL-8, TNF-α, and IL-10), isolated from LPS-induced inflammation, were significantly upregulated (IL-6, IL-8, TNF-α) and downregulated (IL-10), respectively, consistent with previous work in intestinal epithelial cells treated with LPS and TNF-α (Yu et al., 2022). Nevertheless, the cytokine secretion was significantly reduced after probiotic treatment, leading to a significant balance between the pro-inflammatory and anti-inflammatory response, in accordance with the findings of Aghamohammad et al. (2022), who demonstrated that Lactobacillus and Bifidobacterium strains could inhibit the pro-inflammatory cytokines IL-6 and TNF-α and increase the production of IL-10, an important cytokine that maintains mucosal immunity.

Notably, this anti-inflammatory action appears to be mediated by short-chain fatty acids such as acetate, propionate, and butyrate derived from (probiotic) metabolism. Short-chain fatty acids (SCFAs), important metabolites produced by the gut microbiota, are known for their anti-inflammatory activity in the intestinal mucosa. Similar to Akhtar et al. (2022) SCFA concentrations were significantly higher after treatment with 20% PCM, and among the SCFA, butyrate levels showed the highest increase, which is well known for maintaining epithelial integrity and reducing inflammation. Such observations are consistent with SCFAs having a signaling role, in which they can act via G-protein-coupled receptors such as GPR41 and GPR43 to regulate inflammatory responses and immune cell function in the intestine and systemically (Sun et al., 2025). These results are consistent with those of Prado et al. (2023), who also showed that SCFAs can influence immune responses by altering cytokine production and stimulating regulatory T cell differentiation. Although GC-MS analysis enabled quantitative characterization of acetate, propionate, and butyrate in the PCM, the conditioned media constitute complex mixtures that may contain additional organic acids, peptides, proteins, extracellular metabolites, and residual components of the bacterial culture medium. Therefore, the observed epithelial, antioxidant, and anti-inflammatory effects cannot be attributed exclusively to the quantified SCFAs. Comprehensive untargeted metabolomic and proteomic profiling was not performed in the present study, which represents an important limitation. Future studies should incorporate broader compositional profiling, metabolite-specific depletion or supplementation experiments, and appropriate medium-matched controls to identify the individual probiotic-derived factors responsible for the observed cellular responses.

Increased concentrations of acetate, propionate, and butyrate were detected in probiotic-conditioned media and were associated with improved epithelial barrier function and reduced inflammatory and oxidative responses, the present findings do not establish a direct causal relationship between SCFAs and these cellular effects. SCFA supplementation controls, selective depletion experiments, and pharmacological or genetic inhibition of SCFA receptors such as GPR41 and GPR43 were not performed. Therefore, the observed biological responses may reflect the combined activity of SCFAs and other bioactive constituents present in PCM rather than an exclusively SCFA-mediated mechanism. The involvement of GPR41/GPR43 signaling should consequently be considered a potential mechanism supported by previous literature rather than a pathway directly demonstrated in this study. Future studies incorporating purified SCFA supplementation, SCFA-depleted PCM, receptor antagonism or knockdown, and downstream signaling analyses will be necessary to establish the specific contribution and causal mechanisms of SCFAs.

One of the major outcomes of this investigation is a reduction in oxidative stress following probiotic therapy, as verified by both ROS levels and Δψm measurements. Our study also confirmed that LPS exposure significantly increased oxidative stress, as reflected by increased ROS levels and mitochondrial dysfunction, resulting in a reduced JC-1 red/green fluorescence ratio (St-Amant and Bergdahl, 2023). This observation also indicates that probiotic treatment reduced ROS production and helped restore mitochondrial membrane potential, thus counteracting the oxidative damage (Hsu et al., 2023). Our results also suggest a role for probiotics in modulating mitochondrial dysfunction via antioxidant enzyme activity of SOD and catalase, which, in turn, could reduce ROS and help protect cells from oxidative insult (Viana et al., 2025).

Interestingly, probiotics’ antioxidant properties may also be related to the SCFAs (Short-chain fatty acids) they produce (Thananimit et al., 2022). Although it has been previously proposed that SCFAs can activate Nrf2 (nuclear factor erythroid 2-related factor 2), an important transcription factor in the antioxidant response (Cuciniello et al., 2023). We further confirm these results by observing that probiotic-treated groups showed significantly increased antioxidant enzyme activity, suggesting that these probiotics relieve oxidative stress and protect epithelial cells from inflammatory damage (Dashtbanei and Keshtmand, 2023).

We further validate the therapeutic potential of probiotics by regulating oxidative stress, maintaining intestinal barrier integrity, and modulating the inflammatory response to support mucus clearance, both bronchially and systemically, and to influence cellular processes associated with gut–lung communication (Frey et al., 2023). These findings corroborate previous studies indicating that probiotics effectively treat gut dysbiosis and lung inflammation-related diseases associated with the gut-lung axis, such as asthma, COPD, and inflammatory bowel diseases (Chen et al., 2025). Finally, our results contribute to recent advances on probiotics and their metabolites (SCFAs) as a possible, natural way to restore gut integrity and, accordingly, modulate immune responses to diseases associated with a gut-lung axis.

Caco-2 cells were selected because differentiated monolayers develop polarized epithelial characteristics and functional tight junctions suitable for reproducible assessment of TEER, permeability, and barrier-associated responses. A549 cells were employed as a complementary pulmonary epithelial model for controlled assessment of inflammatory, oxidative, and mitochondrial responses, while THP-1-derived macrophages provided an additional immune component. Together, these models provide a reductionist and experimentally controlled platform for investigating intestinal and pulmonary cellular responses potentially relevant to gut–lung communication. Nevertheless, because Caco-2 and A549 are transformed, non-neonatal cell lines and THP-1-derived macrophages represent only a limited immune component, the system does not fully reproduce neonatal gut–lung physiology; this limitation is considered when interpreting the findings. Consequently, the pulmonary findings should be interpreted as mechanistic *in vitro* epithelial responses rather than as direct evidence of neonatal airway physiology or barrier function. Future studies should validate these observations using complementary models, including BEAS-2B cells, primary human bronchial epithelial cells, neonatal airway epithelial cells, and, where feasible, differentiated air–liquid interface cultures or airway organoids that more closely reproduce the physiological characteristics of the respiratory epithelium.

The present study was designed as a mechanistic *in vitro* investigation relevant to the neonatal gut–lung axis rather than as a direct neonatal experimental model. Caco-2, A549, and THP-1 cells were employed to establish a controlled intestinal–pulmonary epithelial and immune interaction system and to characterize probiotic-mediated changes in epithelial barrier integrity, inflammatory signaling, oxidative stress, and metabolite-associated responses. Although these established cell lines provide reproducible platforms for investigating fundamental mechanisms of gut–lung communication, they do not fully reproduce the developmental, cellular, and immunological characteristics of neonatal tissues. Accordingly, the findings should be interpreted as mechanistic evidence supporting biological pathways potentially relevant to neonatal gut–lung homeostasis rather than as direct evidence of neonatal physiological responses. Future studies using neonatal-derived primary intestinal and airway epithelial cells, neonatal organoids, and appropriate neonatal animal models are warranted to validate the translational relevance of these findings to newborn physiology. However, the present experiments were conducted using established cell-line models and therefore cannot establish therapeutic efficacy, clinical safety, disease prevention, or treatment effectiveness. Although alterations in epithelial integrity, inflammation, and oxidative stress are relevant to disorders such as asthma, chronic obstructive pulmonary disease, and inflammatory bowel disease, the current findings should not be directly extrapolated to these clinical conditions. Further validation using primary cells, physiologically relevant co-culture systems, organoids, and appropriate animal models, followed by controlled clinical studies, is required to determine whether the observed cellular effects have translational or therapeutic relevance.

The transwell co-culture system provides an *in vitro* framework for investigating potential soluble mediator-mediated interactions between intestinal and pulmonary compartments; however, the directional transfer of specific metabolites, cytokines, or other signaling molecules between these compartments was not directly quantified or traced. Therefore, the observed improvements in epithelial barrier integrity, inflammatory regulation, oxidative stress, and mitochondrial function are interpreted primarily as *in vitro* cellular responses that may represent mechanisms relevant to gut–lung communication. Further studies incorporating compartment-specific cytokine profiling, SCFA tracing, conditioned-medium transfer experiments, and sequential or bidirectional co-culture approaches are required to establish causal signaling from the intestinal compartment to the pulmonary compartment and vice versa. A limitation of the present study is that the probiotic microorganisms were characterized primarily at the species level. Because probiotic activities may vary among strains of the same species, the observed effects should not be generalized to all strains of *L. rhamnosus*, *B. longum*, or *L. acidophilus*. Future studies should employ fully authenticated strains with strain-level molecular identification and recognized culture-collection accession information to strengthen reproducibility and enable strain-specific interpretation.

The article highlights the promise of probiotics as therapeutic candidates capable of influencing epithelial and inflammatory pathways potentially associated with gut–lung communication. Probiotics represent a novel approach to reduce inflammation-induced epithelial injury by enhancing SCFA production and modulating key signaling pathways. Further studies are warranted to examine the individual mechanisms of action of probiotics and their metabolites, as well as a holistic gut-lung health perspective on interactions between the host immune system and the gut microbiota.

## 5 Conclusion

The present *in vitro* study demonstrates that probiotics and probiotic-conditioned media influence epithelial barrier integrity, inflammatory signaling, oxidative stress, and mitochondrial function in Caco-2 and A549 cell models. Probiotic treatment was associated with increased tight junction-related markers, reduced pro-inflammatory and oxidative responses, and increased concentrations of SCFAs in conditioned media. However, these findings represent cellular responses observed under controlled *in vitro* conditions and should not be interpreted as evidence of clinical efficacy or therapeutic safety. In particular, the present study does not demonstrate that probiotic supplementation can prevent or treat asthma, COPD, inflammatory bowel disease, or other inflammatory disorders. The findings instead provide a mechanistic basis for further investigation of probiotic-associated effects relevant to intestinal and pulmonary epithelial responses. Validation in primary and neonatal-derived cells, advanced co-culture or organoid systems, appropriate animal models, and ultimately well-designed clinical studies will be necessary to establish physiological relevance, safety, and potential therapeutic applicability.

## Data Availability

The original contributions presented in the study are included in the article/supplementary material. Further inquiries can be directed to the corresponding author.
